# Therapeutic effectiveness of anlotinib combined with etoposide in extensive-stage small-cell lung cancer: a single-arm, phase II trial

**DOI:** 10.1007/s10637-023-01398-9

**Published:** 2023-10-14

**Authors:** Yuan Wu, Xuefeng Zhou, Weiqing Zhao, Qiong Wang, Zhengxiang Han, Lifeng Wang, Wenjie Zhou, Tong Zhou, Haizhu Song, Yong Chen, Kaihua Yang, Lin Shi, Banzhou Pan, Renhong Guo, Guoren Zhou, Feng Jiang, Jifeng Feng, Bo Shen

**Affiliations:** 1 Department of Medical Oncology, Jiangsu Cancer Hospital & Jiangsu Institute of Cancer Research & The Affiliated Cancer Hospital of Nanjing Medical University, Nanjing, 210009 China; 2 Department of Oncology, Dongtai People’s Hospital, Dongtai, 224200 China; 3 Department of Oncology, The Third Affiliated Hospital of Soochow University, Suzhou, 213003 China; 4 Department of Oncology, The Jiangyin Clinical College of Xuzhou Medical University, Jiangyin, 214400 China; 5 Department of Oncology, The Affiliated Hospital of Xuzhou Medical University, Xuzhou, 221004 China; 6 The Comprehensive Cancer Center of Nanjing Drum Tower Hospital, Nanjing Drum Tower Hospital, Nanjing, 210008 China; 7 Department of Medical Oncology, Changzhou Cancer Hospital, Changzhou, 213001 China; 8 Department of Medical Oncology, Jinling Hospital, Nanjing, 210016 China; 9 Department of Medical Oncology, Affiliated Hospital of Yangzhou University, Yangzhou, 225001 China; 10 Department of Radiotherapy and Oncology, Affiliated Hospital of Jiangnan University, Wuxi, 214122 China; 11 Department of Thoracic Surgery, The Affiliated Cancer Hospital of Nanjing Medical University & Jiangsu Cancer Hospital & Jiangsu Institute of Cancer Research, Nanjing, 210009 China; 12 42 Baiziting, Xuanwu District, Nanjing, Jiangsu Province China

**Keywords:** Small-cell lung cancer, Anlotinib, Etoposide, Maintenance therapy, Prognosis, Survival

## Abstract

**Background:**

Anlotinib plus chemotherapy as first-line treatment for extensive-stage small-cell lung cancer (ES-SCLC) achieves good efficacy, but there is still room for improvement. This clinical study examined the effectiveness of anlotinib plus etoposide for maintenance therapy in ES-SCLC.

**Methods:**

The current single-arm, prospective phase II study was performed at Jiangsu Cancer Hospital (March 2019 to March 2022). After successful primary etoposide-based therapy, anlotinib was administered at 12 mg/day on days 1 to 14 of 21-day cycles until disease progression or consent withdrawal. All patients also received etoposide at 50 mg/day on days 1 to 14 of 21-day cycles for a maximum of six cycles. Progression-free survival (PFS) constituted the primary study endpoint. Secondary endpoints were overall survival (OS), objective remission rate (ORR), disease control rate (DCR), and safety. In addition, adverse events (AEs) were assessed.

**Results:**

Twenty-eight patients were treated. Median PFS and OS were 8.02 (95%CI 5.36–10.67) and 11.04 (95%CI 10.37–11.68) months, respectively. Totally 9 and 18 participants showed a partial response and stable disease, respectively; ORR and DCR were 32.14% and 96.43%, respectively. The commonest all-grade AEs were fatigue (n = 11, 39.28%), hypertension (n = 11, 39.28%), loss of appetite (n = 9, 32.14%), oral mucositis (n = 7, 25.00%) and proteinuria (n = 6, 21.40%). Grade 3–4 AEs included fatigue (n = 4, 14.28%), hypertension (n = 2, 7.14%), hand and foot syndrome (n = 2, 7.14%), oral mucositis (n = 1, 3.57%), hemoptysis (n = 1, 3.57%), proteinuria (n = 1, 3.57%), gingival bleeding (n = 1, 3.57%), and serum creatinine elevation (n = 1, 3.57%).

**Conclusion:**

Maintenance anlotinib plus etoposide achieves promising PFS and OS in clinical ES-SCLC.

**Registration number:**

ChiCTR1800019421.

**Supplementary Information:**

The online version contains supplementary material available at 10.1007/s10637-023-01398-9.

## Introduction

Small-cell lung cancer (SCLC) SCLC features fast doubling time, elevated growth fraction, and extensive metastases in early disease phases, generally showing widespread hematogenous metastases [1]. SCLC cases comprise approximately 15% of all lung cancers [2], and the age-adjusted incidence of SCLC is 6.23 per 100,000 persons in the United States of America [1, 3]. Extensive-stage (ES)-SCLC includes stage IV (any T, any N, and M1a/b) or T3-T4 excluded from the limited-stage disease per the 8th edition American Joint Committee on Cancer (AJCC) [1].

The best SCLC management is based on surgery, chemotherapy, targeted therapy, and radiotherapy [1, 4], but 5-year overall survival (OS) is only 6.3%, including 27.3% for localized disease, 15.6% for regional disease, and 2.8% for metastatic disease [5]. The recommended primary therapies for ES-SCLC encompass carboplatin/cisplatin, etoposide, and atezolizumab/durvalumab, followed by atezolizumab/durvalumab maintenance therapy [1]. Maintenance therapy after the initial first-line treatment is a recognized treatment approach in clinical ES-SCLC [1], but chemotherapy alone is not as effective as maintenance therapy and could have toxicity issues [6]. Although immunotherapy is an effective maintenance therapy for ES-SCLC [7–10], its high cost prevents access for many patients. Hence, novel treatment strategies are required for improving prognosis in ES-SCLC.

The vascular endothelial growth factor (VEGF) receptor (VEGFR) shows high expression in SCLC [11], justifying the use of anti-VEGF/VEGFR antibodies in SCLC. Many trials examined the efficacy of antiangiogenic drug maintenance therapy for ES-SCLC, but the reported outcomes were unsatisfactory, with low objective response rates (ORRs) (no or small difference vs. control arm), poor progression-free survival (PFS) (4.7–9.1 months), poor OS (8.9–12.1 months), and toxicity issues [1, 12–14]. Anlotinib represents a recently developed tyrosine kinase inhibitor (TKI) targeting multiple receptor tyrosine kinases, including VEGFR1-4, platelet-derived growth factor receptor (PDGFR)α/β, fibroblast growth factor receptor (FGFR)1–4, and c-kit [15]. Anlotinib prevents angiogenesis, decreases tumor cell proliferation, and improves the immune tumor microenvironment [15–17]. Anlotinib is an oral drug (more convenient to use than intravenous drugs), whose tolerance profile is favorable [15]. The first-line therapy for ES-SCLC using anlotinib plus chemotherapy achieves good efficacy, with ORRs of 86–90% and median PFS of 6.0-10.3 months [18–20], and there is still room for improvement.

Therefore, the current work aimed to explore the effectiveness of anlotinib plus etoposide for maintenance therapy in clinical ES-SCLC.

## Materials and methods

### Study design

The current single-arm, prospective phase II trial was performed at the Oncology Department of Jiangsu Cancer Hospital between March 2019 and March 2022. The study followed the Declaration of Helsinki (2000), and had approval from the Ethics Committee of Jiangsu Cancer Hospital. Each patient provided signed informed consent. The current trial was registered at the Chinese clinical trial registry (www.chictr.org.cn, ChiCTR1800019421).

### Participants

Inclusion criteria were: (1) 18 to 75 years old; (2) proven with ES-SCLC by histopathological examination; (3) treatment with standard first-line Etoposide-platinum solely chemotherapy, without progression; (4) Eastern Cooperative Oncology Group (ECOG) performance status of 0–1; (5) one or more computed tomography (CT) measurable lesions; (6) expected survival of at least 3 months; (7) major organ function indicators meeting the following criteria 7 days before the start of treatment: (a) hemoglobin (Hb) ≥ 90 g/L, (b) absolute neutrophil count (ANC) ≥ 1.5 × 10^9^/L, (c) platelets (PLT) ≥ 80 × 10^9^/L, (d) total bilirubin (TBIL) ≤ 1.5 fold the upper limit of normal range (ULN), (e) alanine (ALT) and aspartate (AST) aminotransferase levels ≤ 2.5×ULNs (ALT and AST ≤ 5×ULNs in patients with liver metastasis), (f) serum creatinine (Cr) ≤ 1.5×ULN or creatinine clearance (CCr) ≥ 60 ml/min, and (g) Doppler ultrasound evaluation showing left ventricular ejection fraction (LVEF) ≥ 50%; 7) contraceptive measures in patients of child-bearing age (female patients and female companions of male patients).

Exclusion criteria were: (1) presence of tumor types other than SCLC and mixed-SCLC; (2) a history of severe allergy or allergic constitution; (3) pregnant or breastfeeding women; (4) participation in other clinical trials; (5) pleural effusion or ascites that induced respiratory syndrome (CTCAE grade ≥ 2 dyspnea); (6) symptomatic brain metastases or symptoms controlled for < 2 months; (7) severe and/or uncontrolled diseases such as (a) suboptimal blood pressure control (systolic [SBP] and diastolic [DPB] blood pressure ≥ 150 and ≥ 100 mmHg, respectively), (b) grade ≥ 1 myocardial ischemia or infarction, arrhythmia (QTc ≥ 480 ms), or NYHA grade ≥ 2 congestive heart failure, (c) active or uncontrolled severe infection (CTCAE grade ≥ 2), (d) liver cirrhosis, decompensated liver disease, active hepatitis, or chronic hepatitis that required antiviral treatment, (e) renal failure that required dialysis, (f) a history of immunodeficiency diseases, i.e., HIV or other acquired or congenital immunodeficiency disease, or organ transplantation history, (g) suboptimal control of diabetes (fasting blood glucose (FBG) above 10 mmol/L), (h) urine routine examination showed urine protein ≥++ and 24 h urine protein above 1.0 g, or (i) neurological disease, such as epilepsy, dementia, severe depression, or mania; 8) major surgery, open biopsy, or substantial traumatic injuries within 28 days before inclusion; 9) imaging examination showing tumor invasion of the tissues surrounding vital blood vessels, or a tumor highly possibly invading vital blood vessels; 10) any grade of bleeding constitution or bleeding history, with any grade ≥ 3 bleeding or hemorrhagic events, or nonunion trauma, ulcer, or bone fracture; 11) atrial/venous embolism events within the past 6 months, including cerebrovascular events (e.g., transient ischemic attacks), deep venous thrombosis, or pulmonary embolism; 12) previous psychotropic drug abuse and incapacity of quitting, or with mental diseases; or 13) dysphagia or diagnosed drug absorption disorder.

### Intervention

All patients underwent baseline imaging assessment after enrollment. The participants started treatment within 3 weeks (21 calendar days) from screening. All participants were administered anlotinib plus etoposide. Specifically, all participants were treated with oral anlotinib 12 mg q.d. on days 1–14 of 21-day cycles. Three weeks (21 d) were considered as one cycle. Anlotinib was continually administered until disease progression, consent withdrawal, or intolerable toxicity. The participants were also treated with oral etoposide 50 mg on days 1 to 14 of 21-day cycles. Etoposide treatment lasted for six cycles maximum.

During treatment, imaging examinations were performed every 2 cycles to assess clinical efficacy according to RECIST 1.1 criteria, including complete remission (CR), partial remission (PR), stable disease (SD), and progressive disease (PD). Safety were evaluated every 3 weeks (21 ± 7 days) until disease progression, consent withdrawal, loss to follow-up, or intolerable toxicity. In case of disease progression, the participants were included in the survival follow-up phase, in which follow-up was performed every 56 ± 7 days until death, loss to follow-up, or consent withdrawal. Anti-tumor therapy after disease progression was decided by the investigators. Follow-up was recommended, and patient data were recorded.

Medication was discontinued in case of disease progression. In case of grade 3 or 4 adverse effects, the oral dose of anlotinib was lowered to the next dose level. In patients using a starting dose of 12 mg/day, 10 mg/day and then 8 mg/day were subsequently used. If the initial dose was 10 mg/day, it was lowered to 8 mg/day. No dose lowering was allowed after 8 mg/d; in such cases requiring dose adjustment, treatment discontinuation was applied. When the initial dose was 8 mg/day, treatment discontinuation was directly applied if dose adjustment was necessary.

### Endpoints

The primary endpoint was PFS, which was the time elapsed from the start of therapy to first disease progression as judged by the investigators or per imaging findings or death. Secondary endpoints encompassed OS, ORR, disease control rate (DCR), and safety. OS represented the time elapsed from the start of therapy to death. Objective Response Rate (ORR) was defined as (CR + PR) / (CR + PR + SD + PD) × 100%, and Disease Control Rate (DCR) was defined as (CR + PR + SD) / (CR + PR + SD + PD) × 100%. Treatment-emergent adverse events (TEAEs) were evaluated on the basis of the Common Terminology Criteria for Adverse Events (CTCAE) 4.0.

### Sample size

Previous data revealed a PFS of 2 months in ES-SCLC without maintenance therapy following standard first-line etoposide chemotherapy [21]. The anticipated PFS of patients after anlotinib plus etoposide was 5.5 months. With α = 0.05 and β = 0.2 (power = 80%), 24 participants were required in the present trial. Considering a possible loss to follow-up of 10%, 27 participants were required.

### Statistical analysis

The intent-to-treat (ITT) set encompassed all participants administered the treatment. The per-protocol analysis (PP) set included all participants with high compliance with the study protocol and strictly completed the trial processes per the study protocol. All participants in the PP set completed the drug therapy throughout the study according to the protocol.

Data were analyzed with SPSS 22.0 (SPSS, USA). Normally and skewedly distributed continuous variables were presented as mean ± standard deviation and median (range), respectively. Categorical variables were presented as n (%). Kaplan-Meier curve analysis was utilized to estimate PFS and OS.

## Results

### Characteristics of participants

**Fig. 1:**
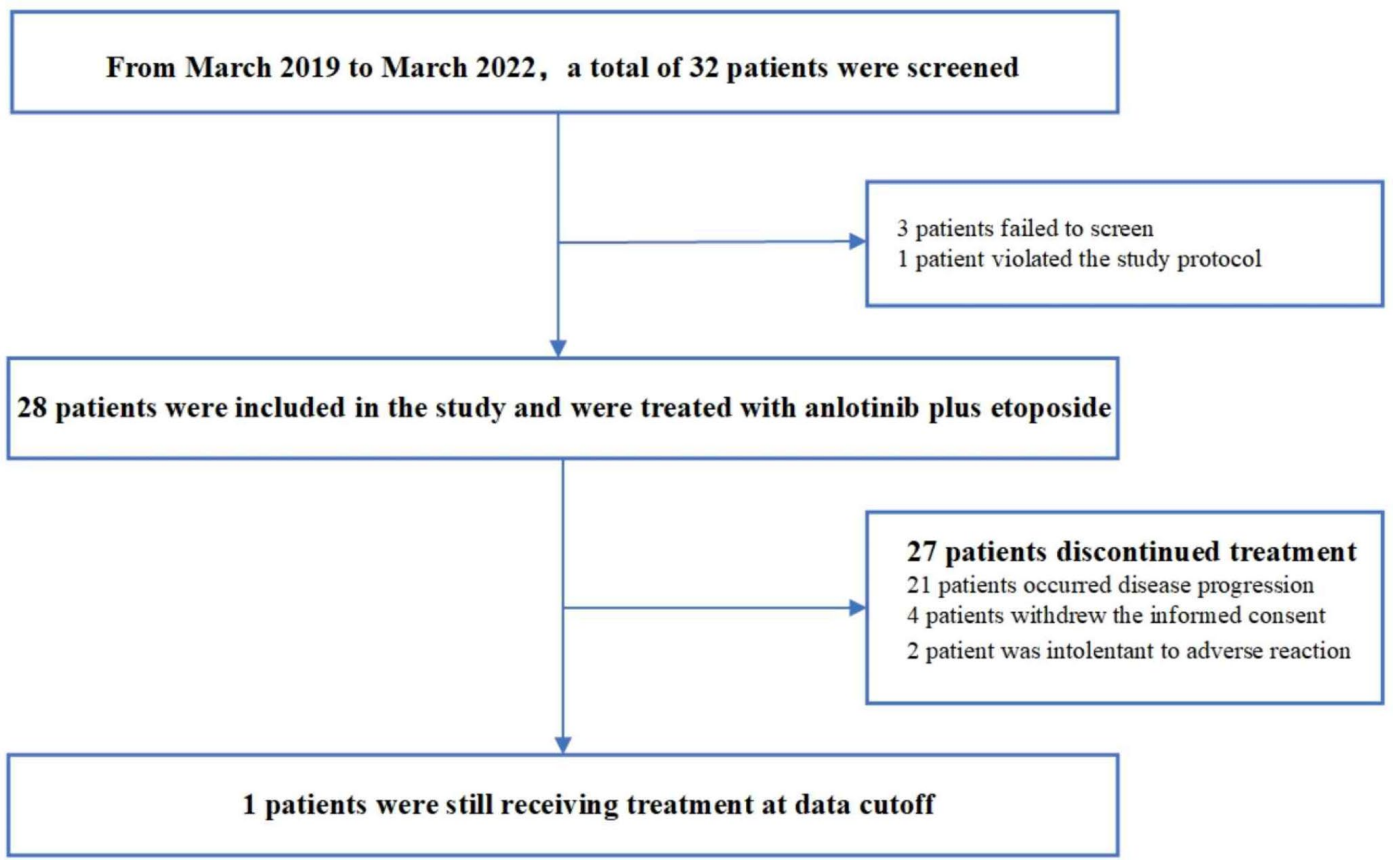
Study flowchart

From March 2019 to March 2022, eligibility screening was conducted for 32 advanced SCLC cases with no progression through first-line therapy (Fig. 1). Cases were excluded for laboratory test failure (n = 3) or protocol violation (n = 1). Finally, 28 patients were included. Table 1 summarizes baseline patient data. Mean age was 66 (38–75) years, and 96.42% were male. Brain metastases were found in 6 (21.43%) participants. All 6 patients with brain metastases were detected before the initiation of etoposide-platinum chemotherapy. The first-line primary therapies were etoposide + cisplatin in 12 (42.86%) participants and etoposide + carboplatin in 16 (57.14%).

**Table 1:** Baseline characteristics of the participants

Clinical characteristic	ITT set (n = 28)	PP set (n = 21)
Age (years)	66 (38–75)	67 (51–75)
Sex, n (%)		
Male	27 (96.42%)	20 (95.23%)
Female	1 (3.57%)	1 (4.76%)
ECOG PS score		
0	3 (10.71%)	1 (4.76%)
1	25 (89.28%)	20 (95.23%)
Pathological type, n (%)		
ES-SCLC	28 (100%)	21 (100%)
Accompaniment of brain metastasis, n (%)		
Yes	6 (21.43%)	4 (19.05%)
No	22 (78.57%)	17 (80.95%)
First-line chemotherapy regimen, n (%)		
Etoposide + cisplatin	12 (42.86%)	10 (47.62%)
Etoposide + carboplatin	16 (57.14%)	11 (52.38%)
Lasting time of chemotherapy (days)		
<90	14 (50%)	11 (52.38%)
>90	14 (50%)	10 (47.62%)
Cycles of chemotherapy		
4	13 (46.43%)	10 (47.62%)
>4	15 (53.57%)	11 (52.38%)
Time from first-line etoposide treatment completed to combination therapy (days)		
<30	14 (64.29%)	8 (38.1%)
≥30	14 (35.71%)	13 (61.9%)
Complication, n (%)		
Diabetes	3 (10.71%)	3 (14.29%)
Hypertension	12 (42.86%)	10 (47.62%)

ITT: intent-to-treat; PP: per-protocol; ECOG PS: Eastern Cooperative Oncology Group Performance Status; ES-SCLC: extensive-stage small cell lung cancer

### Efficacy

**Fig. 2:**
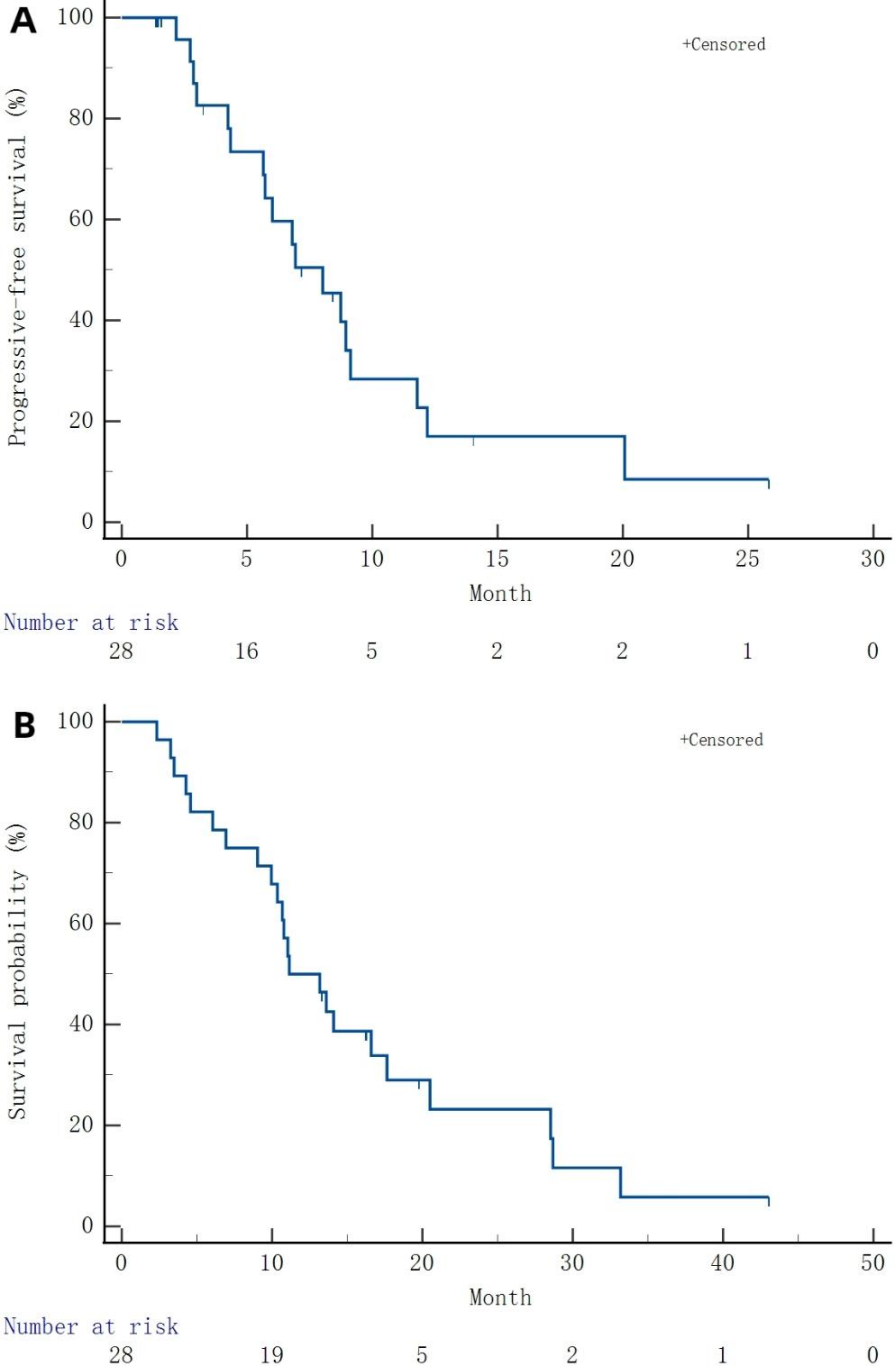
(**A**) Kaplan-Meier curve analysis of progression-free survival (PFS). (**B**) Kaplan-Meier curve analysis of overall survival (OS)

**Fig. 3:**
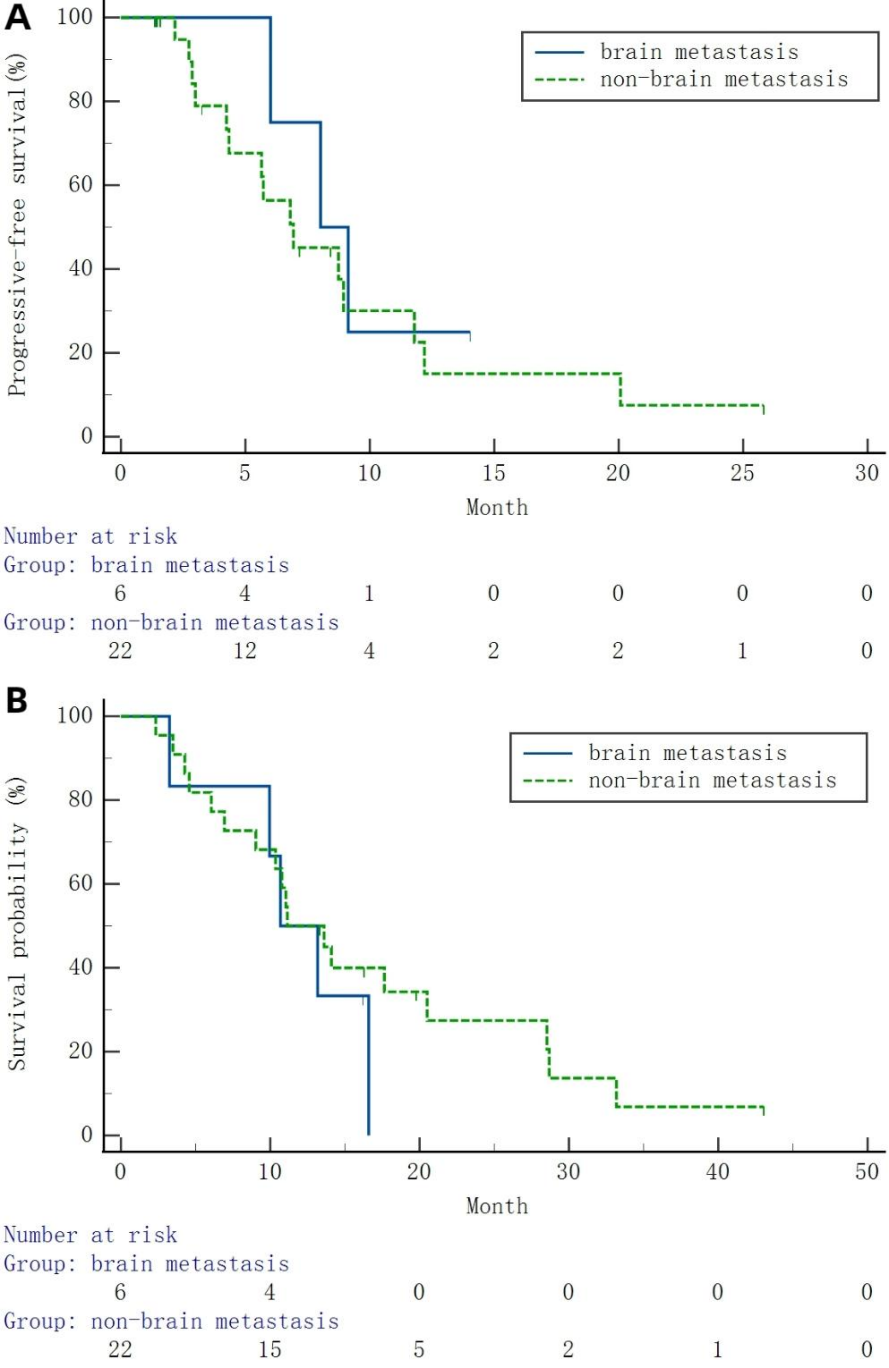
(**A**) Kaplan-Meier curve analysis of progression-free survival (PFS) in the brain metastasis and non-brain metastasis groups. (**B**) Kaplan-Meier curve analysis of overall survival (OS) in the brain metastasis and non-brain metastasis groups

Median PFS was 8.02 (95%CI 5.36–10.67) months (Table 2; Fig. 2A). Median OS was 11.14 (95%CI 7.56–14.72) months (Table 2; Fig. 2B). In the ITT, PR was observed in 9 participants and SD in 18; ORR and DCR were 32.14% and 96.43%, respectively. Based on the PP set, PR was observed in 9 participants and SD in 11; ORR and DCR were 42.86% and 95.24%, respectively (Table 2 and Supplementary Figure S1). In the brain metastasis subgroup, median PFS was 8.02 (95%CI 4.96–11.08) months, and OS was 10.68 (95%CI 6.82–14.55) months (Fig. 3A). In the non-brain metastasis subgroup, median PFS and OS were 6.93 (95%CI 3.24–10.62) and 11.14 (95%CI 6.96–15.32) months, respectively (Fig. 3B).

**Table 2:** Clinical outcomes

Clinical information	ITT set (n = 28)	PP set (n = 21)
mPFS, months (95%CI)	8.02 (5.36–10.67)	
mOS, months (95%CI)	11.14 (7.56–14.72)	
CR, n	0	0
PR, n	9	9
SD, n	18	11
ORR, %	32.14%	42.86%
DCR, %	96.43%	95.24%

ITT: intent-to-treat; PP: per-protocol; mPFS: median progression-free survival; mOS: median overall survival; CR: complete response; PR: partial response; SD: stable disease; ORR: objective response rate; DCR: disease control rate

### Safety

Almost all participants (96.43%) experienced any-grade TEAEs. Any-grade TEAEs leading to anlotinib dose modification were observed in 35.71% of patients. TEAEs leading to anlotinib dose interruption were observed in 14.29% of cases. TEAEs leading to etoposide dose modification were observed in 7.14% of participants (Table 3). The commonest any-grade TEAE was proteinuria (57.14%), followed by anorexia (42.86%), neutropenia (35.71%), asthenia (32.14%), thrombocytopenia (28.57%), hypertension (28.57%), anemia (25.00%), and hand-foot syndrome (25.00%) (Table 3).

**Table 3:** Summary of TEAEs in the ITT set (n = 28)

Adverse events, n (%)	n = 28	
	Any grade	Grade 3–4
TEAEs	27 (96.43)	12 (42.85)
TEAEs leading to anlotinib dose modification	10 (35.71)	8 (28.57)
TEAEs leading to Anlotinib interruptions	4 (14.29)	2 (7.14)
TEAEs leading to etoposide dose modification	2 (7.14)	2 (7.14)
TEAEs leading to Etoposide interruptions	1 (3.57)	1 (3.57)
Proteinuria	16 (57.14)	0 (0)
Anorexia	12 (42.86)	0 (0)
Neutrophil counts decreased	10 (35.71)	3 (10.71)
Asthenia	9 (32.14)	2 (7.14)
Thrombocytopenia	8 (28.57)	1 (3.57)
Hypertension	8 (28.57)	0 (0)
Anemia	7 (25)	0 (0)
Hand-foot syndrome	7 (25)	4 (14.28)
Canker sore	5 (17.86)	1 (3.57)
Creatinine elevation	4 (14.29)	1 (3.57)
Diarrhea	3 (10.71)	0 (0)
Gingival bleeding	3 (10.71)	1 (3.57)
Arthrodynia	2 (7.14)	0 (0)
Pharyngalgia	2 (7.14)	0 (0)
Hemoptysis	1 (3.57)	1 (3.57)

ITT: intent-to-treat; TEAE: treatment emergent adverse events

Grade 3–4 TRAEs were observed in 42.85% of participants. Grade 3–4 TEAEs leading to anlotinib dose modification were observed in 28.57% of cases. TEAEs leading to anlotinib dose interruption were observed in 7.14% of patients. TEAEs leading to etoposide dose modification were observed in 7.14% of participants (Table 3). The commonest grade 3 TRAEs included hand-foot syndrome (14.28%), neutropenia (10.71%), and asthenia (7.14%). In this study, there were no Grade 5 TEAEs, and there were no treatment-related deaths.

## Discussion

This work examined the effectiveness of anlotinib combined with etoposide for maintenance treatment of ES-SCLC cases. The above findings suggest that maintenance treatment with anlotinib and etoposide after primary treatment with etoposide + platinum was effective and safe in clinical ES-SCLC.

Patient survival in ES-SCLC after treatment with etoposide and platinum is poor. Indeed, in a study by Paz-Ares et al. [8] up to six cycles of first-line therapy with platinum and etoposide yielded a median OS of 10.3 months. Roviello and colleagues [21] reported that standard first-line etoposide chemotherapy without maintenance yielded a PFS of 2 months in ES-SCLC. Adding etoposide maintenance following platinum and etoposide first-line therapy does not fare much better. In a phase II trial of four cycles of platinum with etoposide with subsequent etoposide maintenance, median PFS and OS were 9 and 14 months, respectively [22]. Zhang and collaborators [23] compared six cycles of platinum and etoposide followed or not by oral etoposide maintenance therapy and reported that etoposide maintenance improved PFS compared with no maintenance (8.9 vs. 5.9 months), while OS times were similar in both groups (15.0 vs. 14.3 months). Additionally, a meta-analysis by Zhou and colleagues [6] of SCLC supported a lack of efficacy for maintenance chemotherapy in ESCLC cases without statistically significant effects on OS (HR = 0.87, 95%CI 0.71–1.06) or PFS (HR = 0.87, 95%CI 0.62–1.22) compared with the control arm.

Although VEGF is expressed in SCLC, the anti-VEGF antibody bevacizumab yielded conflicting or disappointing results, with ORRs of 58.0-91.9 and median PFS of 4.7–7.8 months [13, 24–26], without differences compared with the control arm [13]. Hence, bevacizumab maintenance therapy is not recommended for SCLC [1]. Classical TKIs (e.g., sorafenib, sunitinib, and pazopanib) are not recommended in SCLC [1] because of a lack of efficacy or high toxicity [27–29]. Sorafenib showed ORRs of 2–11% and median OS times of 5.3–6.7 months in ES-SCLC cases previously administered a maximum of one line of platinum-based therapy [27]. Sunitinib showed a median PFS of 3.7 months, with 19% cases with grade 3 fatigue [28]. In patients responding to primary etoposide plus platinum, adding pazopanib led to maintenance of 3.8 months vs. 1.8 months for placebo [29]. Apatinib might improve PFS (7.8 vs. 4.9 months) and OS (12.1 vs. 8.2 months) in ES-SCLC cases [30]. Another trial of apatinib revealed an ORR of 50% and a median PFS of 3.7 months [31].

Third-line anlotinib monotherapy showed promising efficacy in ES-SCLC cases with/without brain metastasis in the ALTER1202 trial [32, 33]. Following the demonstration of anlotinib monotherapy as a third-line treatment option for SCLC, anlotinib was examined as a first-line therapy in combination with other anticancer drugs. Previous studies of first-line platinum plus etoposide plus anlotinib followed by anlotinib ± etoposide maintenance have reported PFS times of 6.0-10.8 months and OS times of 14.0-17.1 months [18–20]. Still, these three studies used anlotinib in the initial treatment in combination with etoposide and platinum. Exposing patients too early to anlotinib might increase the risk of anlotinib resistance since SCLC has a high rate of treatment resistance due to rapid cell proliferation [34]. Instead, in the present study, anlotinib was administered with etoposide as maintenance treatment in cases already responding to platinum with etoposide. By doing so, exposure to anlotinib is shorter, and treatment costs are lower. This also allows observing the effect of anlotinib without interference from platinum. In this work applying first-line treatment with platinum and etoposide followed by etoposide and anlotinib maintenance, median PFS and OS were 8.0 and 11.1 months, respectively, which are apparently higher than reported for etoposide maintenance [22, 23] but corroborated a previous clinical study of SCLC cases administered etoposide plus platinum followed by anlotinib maintenance (median PFS and OS of 7.7 and 11.0 months, respectively) [35]. Those numbers appeared to be closer to immunotherapy maintenance in ES-SCLC cases, with ORRs of 60–74% and median PFS times of 5.2–13.0 months [7–10]. Unfortunately, immunotherapy is expensive, which cannot be afforded by many patients. Hence, anlotinib combined with etoposide for maintenance therapy could be an effective and more affordable option for immunotherapy. Still, comparisons among trials must be taken with caution because of differences in eligibility criteria and clinical characteristics of the study populations. Future trials should be designed to directly compare anlotinib vs. immunotherapy for maintenance treatment in ES-SCLC cases.

In this work, 96.43% of the examined participants experienced any-grade TEAEs, with grade 3–4 TEAEs in 42.85% of cases. Those numbers are lower than reported for ES-SCLC cases administered other TKIs as maintenance treatment in phase II trials. Indeed, maintenance treatment with sorafenib resulted in 23% treatment discontinuation due to AEs [27]. A study of sunitinib maintenance reported a dose reduction in 48% of participants and grade 3–4 AEs in 54% of cases [28]. Pazopanib was also associated with high rates of toxicities, with treatment interruption in 52% of the examined participants [29]. Maintenance with immunotherapy also has safety issues, with any-grade AEs observed in 100% of participants, grade 3–4 AEs in 45–68%, and grade 5 AEs in 5% [7, 8, 10]. Therefore, anlotinib has a safety profile that is at least not worse, and maybe even better, than other TKIs and immunotherapy tested for maintenance therapy in ES-SCLC. In this study, the commonest any-grade TEAEs included proteinuria, anorexia, neutropenia, asthenia, thrombocytopenia, hypertension, anemia, and hand-foot syndrome, without new safety signals [18–20, 36, 37]. These results suggest the tolerability of anlotinib + etoposide for maintenance treatment in ES-SCLC.

This study had multiple limitations. First, this was a phase II trial without a control group. In addition, a single center design was used with small sample size. The recent advent of immunotherapy with platinum and etoposide as primary therapy [1] might require examining anlotinib in future studies.

In conclusion, anlotinib and etoposide is promising and safe in ES-SCLC after primary therapy with etoposide + platinum.

### Electronic supplementary material

Below is the link to the electronic supplementary material.

Supplementary figure S1: Waterfall plot of prognosis in the ITT set

## Data Availability

The data generated or analyzed during this study are included in this published article.

## Declarations

### Animal Studies

N/A.
