# Supplementary figures and images for: Therapeutic effectiveness of anlotinib combined with etoposide in extensive-stage small-cell lung cancer: a single-arm, phase II trial
Source: Invest New Drugs. 2023 Oct 14;41(6):825–33. doi: 10.1007/s10637-023-01398-9 (PMC10663256; doi:10.1007/s10637-023-01398-9)

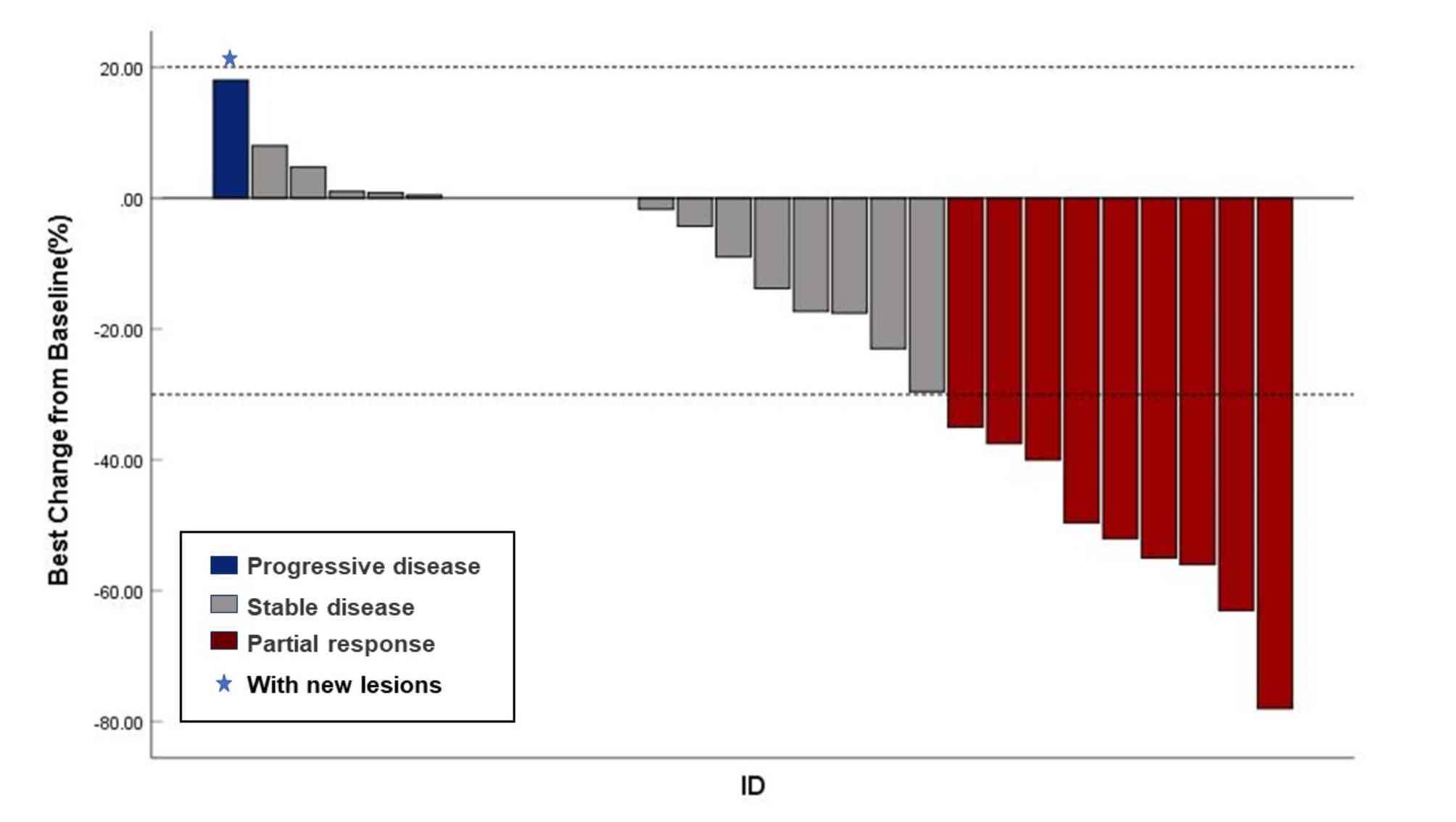

Supplement: Supplementary file 1 — Supplementary figure S1: Waterfall plot of prognosis in the ITT set [file 10637_2023_1398_MOESM1_ESM.jpg]
